# Comprehensive analysis of VEGF/VEGFR inhibitor-induced immune-mediated hypertension: integrating pharmacovigilance, clinical data, and preclinical models

**DOI:** 10.3389/fimmu.2024.1488853

**Published:** 2024-10-22

**Authors:** Hongyu Kuang, Qingkai Yan, Zhanzhi Li, Anqi Lin, Kailai Li, Jian Zhang, Peng Luo, Yuehui Yin

**Affiliations:** ^1^ Department of Cardiology, The Second Affiliated Hospital of Chongqing Medical University, Chongqing, China; ^2^ Department of Cardiology, Bishan Hospital of Chongqing Medical University, Chongqing, China; ^3^ School of Clinical Medicine, Hangzhou Medical College, Hangzhou, Zhejiang, China; ^4^ Department of Oncology, Zhujiang Hospital, Southern Medical University, Guangzhou, Guangdong, China

**Keywords:** vascular endothelial growth factor inhibitors, vascular endothelial growth factor receptor inhibitors, cancer, immune-mediated hypertension, cardiovascular risk

## Abstract

**Introduction:**

This study aimed to elucidate the differential immunological mechanisms and characteristics of hypertension induced by VEGF inhibitors (VEGFi) and VEGF receptor inhibitors (VEGFRi), with the goal of optimizing monitoring strategies and treatment protocols.

**Methods:**

We investigated the risk of immune-related adverse events associated with VEGFi/VEGFRi-induced hypertension by analyzing the FDA Adverse Event Reporting System (FAERS) database. Findings were corroborated with blood pressure characteristics observed in clinical patients and preclinical models exposed to various VEGF/VEGFRi. Clinical and preclinical studies were conducted to compare immunological responses and hypertension profiles between inhibitor classes. An integrative analysis across cancer types and species was performed, focusing on key signaling pathways.

**Results:**

Analysis of FAERS data, in conjunction with clinical observations, revealed that both VEGFi and VEGFRi significantly elevated the risk of immune-mediated, blood pressure-related adverse events (ROR=7.75, 95% CI: 7.76-7.95). Subsequent clinical and preclinical studies demonstrated differential immunological responses and hypertension profiles between inhibitor classes. VEGFRi exhibited a more rapid onset, greater blood pressure elevation, and higher incidence of immune-mediated adverse events compared to VEGFi (Systolic BP: ROR=0 for VEGFi vs. ROR=12.25, 95% CI: 6.54-22.96 for VEGFRi; Diastolic BP: ROR=5.09, 95% CI: 0.60-43.61 for VEGFi vs. ROR=12.90, 95% CI: 3.73-44.55 for VEGFRi). Integrative analysis across cancer types and species, focusing on key signaling pathways, revealed that VEGF/VEGFRi-induced blood pressure elevation was associated with immunomodulation of the mitogen activated protein kinase (MAPK) pathway (R=-0.379, P=0.0435), alterations in triglyceride metabolism (R=-0.664, P=0.0001), modulation of myo-inositol 1,4,5-trisphosphate-sensitive calcium release channel activity (R=0.389, P=0.0378), and dysregulation of nitric oxide eNOS activation and metabolism (R=-0.439, P=0.0179).

**Discussion:**

The temporal dynamics of these effects demonstrated greater significance than dose-dependent responses. Both VEGFi and VEGFRi significantly augmented the risk of immune-mediated, blood pressure-related adverse events, with VEGFRi inducing a more rapid and pronounced onset of blood pressure elevation and a higher incidence of immune-related, blood pressure-associated adverse events compared to VEGFi.

## 1 Introduction

Malignant tumors are one of the major diseases that seriously threaten public health, with a complex pathogenesis resulting from the interaction of multiple factors (1, 2). The combination of various risk factors has led to a year-on-year increase in cancer incidence, with an estimated 19.3 million new cancer cases and nearly 1 million deaths worldwide in 2020 (3, 4), imposing a considerable economic and healthcare burden globally. For early-stage tumors, traditional treatments such as radiotherapy and chemotherapy can achieve eradication or symptomatic relief; however, they often have profound side effects that may destroy normal cells, resulting in decreased immunity and potentially increasing the risk of other tumors (5, 6). Compared to conventional treatment, immunotherapy is relatively less harmful to patients, but only a small percentage of patients can benefit from immunotherapy in the long term (7). Therefore, the search for suitable targeted drugs has become the focus of attention in clinical tumor therapy.

Vascular endothelial growth factor (VEGF) is a highly specific pro-vascular endothelial cell growth factor that promotes increased vascular permeability, extracellular matrix degeneration, vascular endothelial cell migration, proliferation, and angiogenesis. The high-affinity receptor that binds specifically to VEGF is VEGFR, which is mainly classified into three categories: VEGFR-1, VEGFR-2, and VEGFR-3 (8, 9). Current studies have confirmed that the biological role of VEGF extends far beyond its regulation of angiogenesis, as it is overexpressed in the vast majority of tumors and is widely considered to be a key factor mediating tumor angiogenesis (10). Consequently, the core role of the VEGF pathway in tumors makes it a rational target for anti-cancer therapy. Angiogenesis inhibitors targeting any component of the VEGF pathway, including VEGF inhibitors (VEGFi), VEGF receptor inhibitors (VEGFRi), and small molecule complex kinase inhibitors, can inhibit endothelial proliferation and disrupt the vascular supply of nutrients and oxygen, thereby achieving the goal of curbing tumor growth and metastasis (11, 12). Evidence has shown that VEGFi can significantly increase overall survival (OS) (HR=0.83, 95% CI [0.74-0.93]) and progression-free survival (PFS) (HR=0.49, 95% CI [0.40-0.61]) (13). Additionally, evidence-based medicine has demonstrated that the risk of death in patients with metastatic renal cell carcinoma was reduced by 13% (HR=0.87, 95% CI=0.80-0.95) after VEGFi/VEGFRi treatment (14). In patients with nasopharyngeal carcinoma treated with VEGFi/VEGFRi, the objective response rate (ORR) and disease control rate (DCR) were 37% (95% CI [17-60%]) and 70% (95% CI [51-85%]), respectively, with 1-year OS and PFS of 34% and 62% (15).

Although VEGF pathway inhibitors are generally well-tolerated, adverse effects such as anemia, gastrointestinal reactions, bleeding, and proteinuria still exist (16, 17). Moreover, it is very common for inhibitors of the VEGF pathway to induce hypertension (18, 19), which may lead to an enhanced risk of cardiovascular diseases, such as hypertension-associated cerebral hemorrhage, myocardial infarction, and heart failure. These cardiovascular toxicities have harmful implications for patients with cancers, potentially requiring dose adjustments or cessation of therapy. Previously, for cancer patients with limited life expectancy, the monitoring and control of blood pressure (BP) were not prioritized; however, in the last 20 years, cardiovascular mortality induced by anti-cancer drugs has been found to exceed cancer mortality (18, 20). Therefore, long-term monitoring of BP and antihypertensive management is critical for patients treated with VEGF/VEGFRi. Notably, a dose correlation has been identified between the degree of BP elevation and VEGF(R) inhibitors, although evidence of a time dependence is lacking (21, 22). Meanwhile, it remains unclear whether different VEGF pathway inhibitors differ in their effects on BP in cancer patients and how to choose an appropriate treatment regimen by combining the characteristic trends of hypertension induced by VEGF pathway inhibitors.

Therefore, this study utilizes the FAERS database and combines evidence from hospital observations of BP levels after using different types of VEGF/VEGFRi. It further validates the effects and onset time of VEGFi and VEGFRi on BP to provide theoretical support for the monitoring and management of BP in cancer patients receiving VEGF(R) inhibitors during the course of treatment.

## 2 Methods

### 2.1 FEARS

We conducted a pharmacovigilance study of blood pressure-related adverse reactions to inhibitors of VEGF and its receptor (VEGFR) based on the FAERS database (23). The FAERS is a publicly available database of safety reports submitted by patients, healthcare professionals, and pharmaceutical companies. In this study, we specifically focused on the following VEGFi and VEGFRi: VEGFi: Bevacizumab, Ranibizumab, Brolucizumab, Aflibercept, Conbercept, Pegaptanib; VEGFRi: Ramucirumab, Nintedanib, Apatinib, Axitinib, Sunitinib, Sorafenib, Regorafenib, Vandetanib, Cabozantinib, Pazopanib, Lenvatinib, Anlotinib, Fruquintinib, Tivozanib, Cediranib, Brivanib (**Supplementary Table S1**) (24). Using these drug names as keywords, we obtained the reported data from FAERS from January 1, 2013, to December 31, 2023, and screened cases with the primary suspicion of using these VEGFi and VEGFRi for the study.

Adverse reactions reported in the FAERS database were based on the preferred terms (PT) from the Medical Dictionary for Regulatory Activities (MedDRA, version 25.1), which provides a unique description of medical concepts, including signs, symptoms, and disease diagnoses, through its five-level logical structure. We paid special attention to PTs related to “blood pressure” and identified a total of 72 adverse reactions related to blood pressure (**Supplementary Table S2**).

#### 2.1.1 Data processing flow

**Figure 1:**
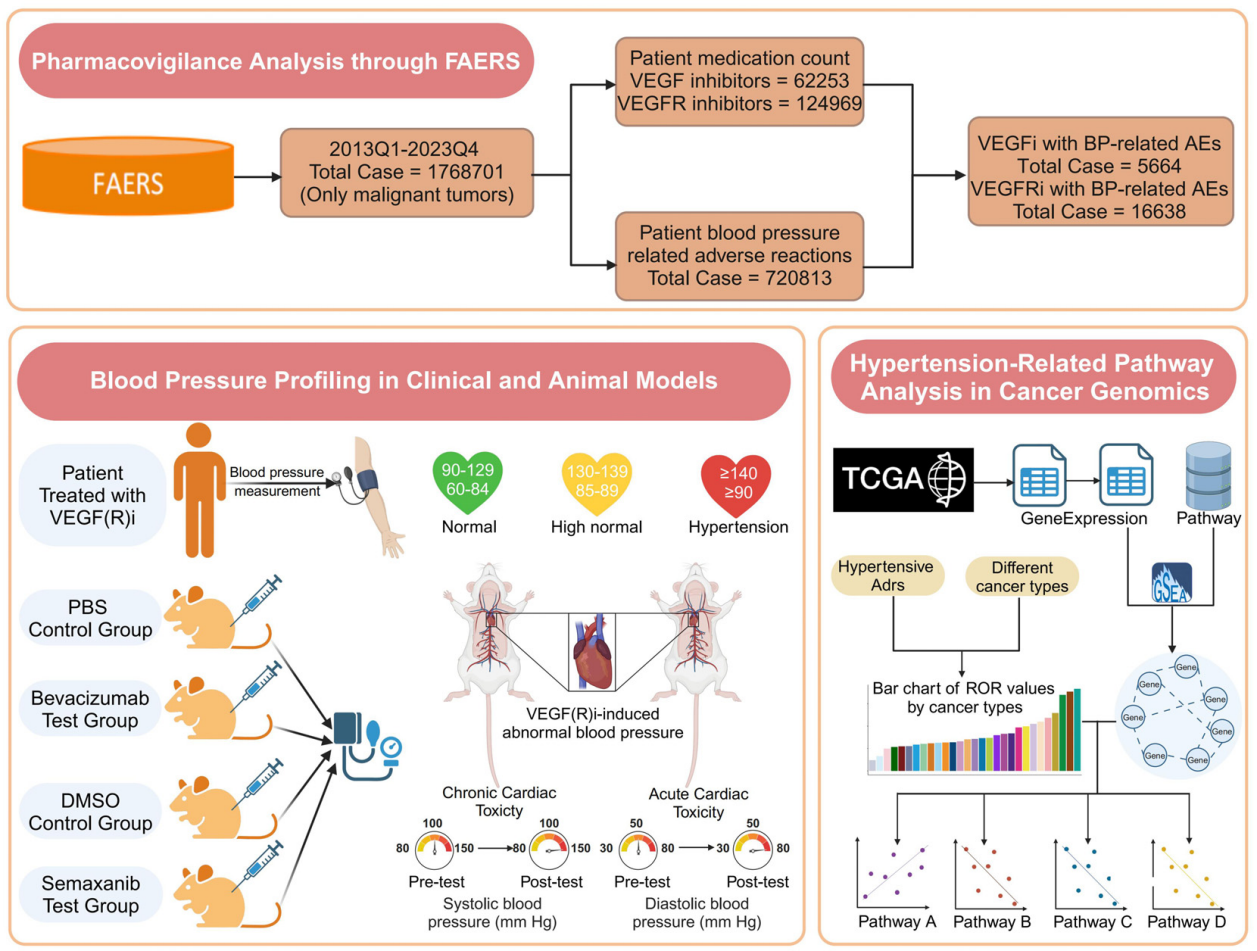
The flowchart illustrates the multidimensional research framework of this paper, which includes monitoring and analysis of drug safety in the FAERS database, monitoring of blood pressure changes in clinical and animal models, and cancer genomics analysis using the TCGA database.

Among the VEGFi- and VEGFRi-related reports obtained from the FAERS database, we first performed the step of removing duplicates by identifying and excluding those reports with identical values in the fields of gender, age, country, date of event, adverse reaction, drug, and indication to ensure uniqueness and accuracy of the data (25). The remaining reports were screened to include only those whose indications were related to malignant diseases, from which 1,768,701 patients with malignant tumors were included. This step was taken to ensure that the focus of the study was on our population of interest and to exclude blood pressure-related adverse events that may have been caused by other factors, such as conditions that may be suggestive of a blood pressure problem in a reported adverse reaction but are not directly related to malignant diseases (26). We identified the patient reports needed for the study and ultimately obtained the overall FAERS database adverse reaction reports used for further analyses. This included 62,253 patients with malignant tumors treated with VEGFi and 124,969 patients with malignant tumors treated with VEGFRi (**Figure 1**).

#### 2.1.2 Signal analysis

In the current study, we focused on VEGFi and VEGFRi and provided a comprehensive analysis of the blood pressure-related adverse events that may occur during treatment with these agents (27). We employed disproportionality analysis, a statistical method frequently used in pharmacovigilance studies, to evaluate the strength of the association between a specific drug and a particular adverse event (28). By comparing the reporting probability of an event of interest for a specific drug to its reporting probability for other drugs in the FAERS database, we were able to identify signals of blood pressure abnormalities associated with VEGFi and VEGFRi. Specifically, we utilized the reporting odds ratio (ROR) to assess the relative risk of a specific adverse event occurring (29), while the statistical significance and strength of these signals were determined by the information component (IC) and its lower limit value (IC025) (30). To perform this analysis, we extracted adverse event reports related to VEGFi and VEGFRi from the FAERS database and generated a comprehensive Adverse Drug Reactions league table, which served as the foundation for the subsequent ROR and IC calculations. The ROR calculation reveals the relative frequency at which a specific adverse event is reported for a particular drug compared to other drugs (31). Additionally, the IC and its lower limit value, IC025, provide further evidence of the signal strength and reliability of these adverse events.

The ROR and its 95% confidence interval (CI) were calculated as follows:


$$\text{ROR}=\frac{\text{a}/\text{c}}{\text{b}/\text{d}}$$

$$95\text{\%CI}={\text{e}}^{\ln\text{ROR }\pm \;1.96\sqrt{\frac{1}{\text{a}}+\frac{1}{\text{b}}+\frac{1}{\text{c}}+\frac{1}{\text{d}}}}$$

The information component (IC) and its lower limit value (IC025) are calculated as follows:


$$\text{IC}=\log_{2}\left(\frac{\text{a}}{\left(\text{a}+\text{b}+\text{c}+\text{d}\right)/\left(\text{a}+\text{b}\right)/\left(\text{c}+\text{d}\right)}\right)$$

$$\text{IC}025={\text{e}}^{\ln\text{IC}-1.96\sqrt{\frac{1}{\text{a}}+\frac{1}{\text{b}}+\frac{1}{\text{c}}+\frac{1}{\text{d}}}}$$

We considered blood pressure-related adverse events to be highly associated with the use of VEGFi and VEGFRi if the number of reports of blood pressure-related adverse events was at least three, and the lower limit of the 95% confidence interval of their ROR was greater than one, and the lower bound of the information component (IC025) was greater than zero. Overall, we included 5,664 patients with malignancies who used VEGFi and experienced blood pressure-related adverse events, and 16,638 patients with malignancies who used VEGFRi and experienced blood pressure-related adverse events.

#### 2.1.3 Analysis of the time to onset of adverse reactions related to blood pressure

We initially compared the onset time of blood pressure-related adverse reactions between VEGFi and VEGFRi. We then selected eight drugs, each with over 1,000 cases, for detailed analysis. Among them, Bevacizumab was identified as a VEGFi, while the other seven drugs—Lenvatinib, Cabozantinib, Sunitinib, Pazopanib, Axitinib, Regorafenib, and Sorafenib—were classified as VEGFRi. Additionally, we identified 12 hypertension-related adverse reactions and thoroughly compared how the selected drugs influenced these reactions.

### 2.2 Analysis of pre- and post-medication blood pressure changes in patients from local hospitals

In our analysis at Zhujiang Hospital of Southern Medical University, we examined how VEGFi and VEGFRi affect diastolic and systolic blood pressure. After a thorough data cleaning process, we included 1,087 patients treated with VEGFi and 529 with VEGFRi (32). The study complied with international and national ethics guidelines and was approved by the Ethics Committee of Zhujiang Hospital of Southern Medical University (33).

To ensure the accuracy of the data, we recorded the maximum systolic and corresponding diastolic blood pressure values before and after the first medication dose, ensuring consistency in measurement. This approach allowed us to capture possible blood pressure maxima and thus more accurately assess the potential impact of the medication on the patient’s blood pressure. In our analysis, we compared the overall diastolic and systolic blood pressure before and after medication. Further, we referred to the 2023 European Guidelines for the Diagnosis of Hypertension (34) and categorized blood pressure as normotensive (<130/85 mmHg), high normotensive (130-139/85-89 mmHg), and hypertensive (>140/90 mmHg). These definitions helped us to categorize the patients’ blood pressure changes into different clinically relevant categories and provide a comprehensive assessment of the changes in blood pressure levels before and after medication (35).

### 2.3 Calculation of enrichment scores for biological pathways in TCGA pan-cancer

To gain a deeper understanding of the adverse effects of VEGFi and VEGFRi associated with blood pressure at the molecular level, we downloaded transcriptome data for 35 cancer types from The Cancer Genome Atlas (TCGA) program via the UCSC Xena database for this study. We converted expression data from Fragments Per Kilobase of transcript per Million mapped reads (FPKM) to Transcripts Per Million (TPM) format (36) and conducted single-sample gene set enrichment analysis (ssGSEA) using the GSVA package (37). For each cancer sample, we calculated enrichment scores of biological pathways using annotated gene sets from the Molecular Signatures Database (MSigDB) (38), including Gene Ontology (GO), Kyoto Encyclopedia of Genes and Genomes (KEGG), and Reactome pathways for the analysis. The score reflects the level of activity of a particular gene set in a biological process, as evidenced by a uniform up- or down-regulation of the member genes. Our study aimed to uncover biological mechanisms linked to blood pressure-related adverse events by analyzing the correlation between the ROR of adverse reactions to VEGFi and VEGFRi and pathway activation levels in different cancers.

### 2.4 Animal experiments

#### 2.4.1 Experimental groups

Forty-eight male C57BL/6J mice, aged 6-8 weeks and weighing 25 g, were purchased from Jiangsu Huachuang Xinnuo Pharmaceutical Technology Co. The experimental procedures were approved by the Ethics Committee of the Animal Experimentation Center of the Second Affiliated Hospital of Chongqing Medical University. Bevacizumab (a VEGF ligand inhibitor, No. 216974-75-3) and Semaxanib (a VEGFRi, 204005-46-9) were purchased from MedChemExpress (MCE). Bevacizumab was solubilized in phosphate-buffered saline (PBS), and Semaxanib was solubilized in dimethyl sulfoxide (DMSO).

Animals were divided into two models according to the randomized numeric table method: the Chronic Cardiac Toxicity (CCT) model (n=24) and the Acute Cardiac Toxicity (ACT) model (n=24). In the CCT model, 24 mice were randomly divided into four groups: PBS (Bevacizumab control, n=6), Bevacizumab (n=6), DMSO (Semaxanib control, n=6), and Semaxanib (n=6). The dose of Bevacizumab was 5 mg/kg (twice per week) (39), and Semaxanib was 10 mg/kg (twice per week) (40) in a volume of 200 μL, administered for 4 weeks. In the ACT model, 24 mice were randomly divided into four groups: PBS (Bevacizumab control group, n=6), Bevacizumab (n=6), DMSO (Semaxanib control, n=6), and Semaxanib (n=6). The dose was double that of the CCT model, i.e., 10 mg/kg (twice per week) for Bevacizumab and 20 mg/kg (twice per week) for Semaxanib in a volume of 200 μL, with an intervention time of 2 weeks.

#### 2.4.2 Blood pressure measurement

Noninvasive tail cuff measurements were used to monitor the blood pressure of mice from all groups at consistent time points to minimize the effect of blood pressure rhythm. Ambient temperature was recorded prior to each measurement. After acclimating the mice, the inflatable tail sleeve was placed at the base of the mouse’s tail, ensuring a close fit with the tail artery. The blood pressure monitoring system was activated once the mice were sufficiently calm, and blood pressure levels were recorded when pulse fluctuation signals appeared on the screen.

### 2.5 Statistical analysis

In this study, the cumulative distribution function (CDF) was employed to plot the timeline of adverse reactions to VEGFi and VEGFRi, visualizing the duration from drug initiation to reaction onset and illustrating the time span from initial drug use to the onset of adverse reactions. The Mann-Whitney U test was used to assess differences in the median onset time of blood pressure-related adverse reactions between VEGFi and VEGFRi (41). Additionally, patient blood pressure data from Zhujiang Hospital of Southern Medical University were analyzed to compare changes before and after drug administration. For animal experiments, the data represented six independent samples, and the results were expressed as mean ± standard error of measurement (SEM) to ensure the accuracy of data analysis. The n value represents the number of biological replicates, emphasizing the biological significance of the experimental repetitions rather than mere technical repetitions. To ensure that the data were normally distributed for accurate statistical analysis, the Shapiro-Wilk normality test was performed for each group, and Student’s t-test was used to compare significant differences between two independent samples. In analyzing the biopathway data from the TCGA pan-cancer project, single-sample gene set enrichment analysis (ssGSEA) and Spearman correlation analysis were applied to reveal the correlation between the level of biopathway activation and drug-induced blood pressure-related adverse effects. All data are presented as mean ± standard error of the mean (SEM), and the specific group size (n) of each experiment is clearly labeled, emphasizing the importance of biological replication. A P-value < 0.05 was considered statistically significant. All data processing, statistical analyses, and visualization of graphs were performed using R software (version 4.3.1, https://www.r-project.org/) and GraphPad Prism 9.0 software.

## 3 Results

### 3.1 Blood pressure-related adverse effects of VEGFi and VEGFRi

In our analysis of the FAERS database, we focused on blood pressure-related adverse reactions associated with VEGFi and VEGFRi therapy (**Supplementary Table S3**). Among these 18 adverse reactions, 11 were directly related to hypertension, suggesting that hypertension was a significant component of these adverse reactions. Actually, ROR for hypertension was 7.75 [7.56-7.95] with an IC of 2.14 [2.11], indicating a strong association with the use of VEGFi and VEGFRi, as evidenced by the stable confidence intervals.

**Figure 2:**
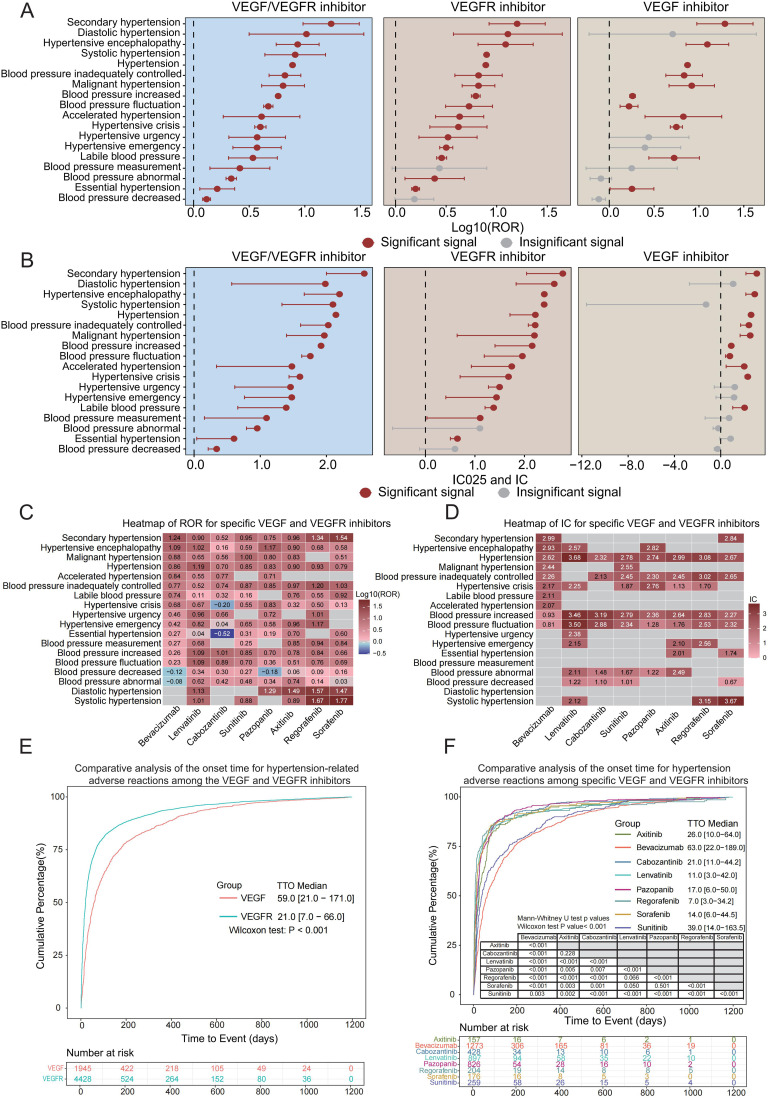
Blood pressure-related ROR, IC, and TTO analysis of VEGFi and VEGFRi. **(A)** Log10 (ROR) comparison of blood pressure-related adverse reactions of VEGFi vs. VEGFRi. **(B)** IC025 and IC comparison of blood pressure-related adverse reactions of VEGFi vs. VEGFRi. **(C)** Heatmap of ROR of specific VEGFi vs. VEGFRi. **(D)** Heatmap of IC of specific VEGFi vs. VEGFRi. **(E)** Comparative analysis of time to onset of blood pressure-related adverse reactions of VEGFi vs. VEGFRi. **(F)** Comparative analysis of time to onset of hypertension adverse reactions induced by specific VEGFi vs. VEGFRi.

Further analysis of these 18 positive adverse reaction signals revealed that both VEGFi and VEGFRi presented multiple signals in blood pressure-related adverse reactions; however, we noted that VEGFi showed fewer positive signals for blood pressure-related adverse reactions compared to VEGFRi (**Figures 2A, B**). Although the use of both classes of drugs should be closely monitored for blood pressure-related markers, VEGFRi had more significant signals in certain adverse reactions. For example, the ROR for diastolic hypertension induced by VEGFRi was 12.90 [3.73-44.55] with an IC of 2.20 [0.64], compared to 5.09 [0.60-43.61] for VEGFi with an IC of 1.08 [-2.71], and the ROR for systolic hypertension induced by VEGFRi was 12.25 [6.54-22.96] with an IC of 2.60 [1.83], compared to 0 for VEGFi, where the IC was -1.27 [-11.59]. These results suggest the need for more stringent blood pressure monitoring of patients receiving VEGFRi at the time of treatment, as such drugs may increase the risk of specific blood pressure-related adverse events.

Our analysis focused on eight VEGFi and VEGFRi, selected based on their usage frequency in the FAERS database for reporting blood pressure-related adverse events, including one VEGFi (bevacizumab) and seven VEGFRi (lenvatinib, cabozantinib, sunitinib, pazopanib, axitinib, regorafenib, and sorafenib). The analysis revealed significant ROR and IC025 values for hypertension across all examined drugs (**Figures 2C, D**), indicating a strong risk signal for blood pressure elevation associated with both VEGFi and VEGFRi.

In particular, compared to the seven VEGFRi, bevacizumab (a VEGF inhibitor) exhibited higher ROR and IC values for conditions such as secondary and malignant hypertension. Lenvatinib and other VEGFR inhibitors demonstrated signals across various adverse categories, suggesting a link to a wider range of blood pressure issues. Sorafenib and sunitinib showed high signal intensity in various adverse categories, indicating their potential impact on diverse blood pressure-related events. Different inhibitors showed different propensities for inducing specific blood pressure abnormalities.

### 3.2 Analysis of time to onset of adverse blood pressure reactions to VEGFi and VEGFRi

When comparing the overall time to onset of hypertension with VEGFi versus VEGFRi, we discovered that the time to onset of blood pressure-related adverse events was significantly shorter with VEGFRi than with VEGFi. Cumulative distribution curve analysis revealed that the group of patients receiving VEGFRi had significantly shorter median times to the onset of hypertension-related adverse reactions compared with the group of patients receiving VEGFi, 21.0 days (IQR 7.0-66.0) and 59.0 days (IQR 21.0-171.0), respectively. The results of the Wilcoxon test (P < 0.001) further indicated that there was a statistically significant difference in time to onset between the two groups (**Figure 2E**, P < 0.05). These findings suggest that in clinical settings, VEGFRi could lead to a quicker onset of blood pressure-related adverse events.

When we compared in-depth the time to onset due to specific VEGFi versus VEGFRi, significant temporal differences in the adverse effects associated with elevated blood pressure caused by these drugs were revealed. Specifically, the median time to onset for bevacizumab was 63.0 days [IQR 21.0-184.8], which was the longest of all the drugs, suggesting that bevacizumab induced blood pressure-related adverse effects later than the VEGFRi. Among the seven VEGFRi, regorafenib had the shortest median onset time of 8.0 days [IQR 3.0-29.0], whereas sunitinib had the longest time of 41.0 days [IQR 14.0-179.0], revealing significant differences in the rate of inducing BP-related adverse effects between the drugs (**Supplementary Figure S1**). The results of the Mann-Whitney U-test showed that all the drugs except comparisons between ramucirumab and regorafenib (P = 0.175) and sorafenib and regorafenib (P=0.148) did not show significance, whereas comparisons between all other drug combinations showed significant differences (all P < 0.05).

In our detailed analysis, we narrowed our focus to 11 out of 18 identified adverse reactions that were directly related to hypertension. This approach helped us precisely evaluate the impact of various drugs on inducing hypertension. This stratification strategy allowed us to accurately assess the impact of different drugs on inducing hypertension. Our analysis reconfirmed that bevacizumab exhibited a significant prolongation of onset time relative to other VEGFRi. Among the VEGFRi analyzed in combination, regorafenib maintained the shortest median time to onset of 7.0 days [IQR 3.0-34.2], while the other drugs showed significant differences in time to onset (**Figure 2F**). Consistent with prior results, the Mann-Whitney U-test validated significant differences in the onset times across drugs, further substantiating the importance of timing in hypertension onset.

### 3.3 Analysis of blood pressure changes in clinical patients

**Figure 3:**
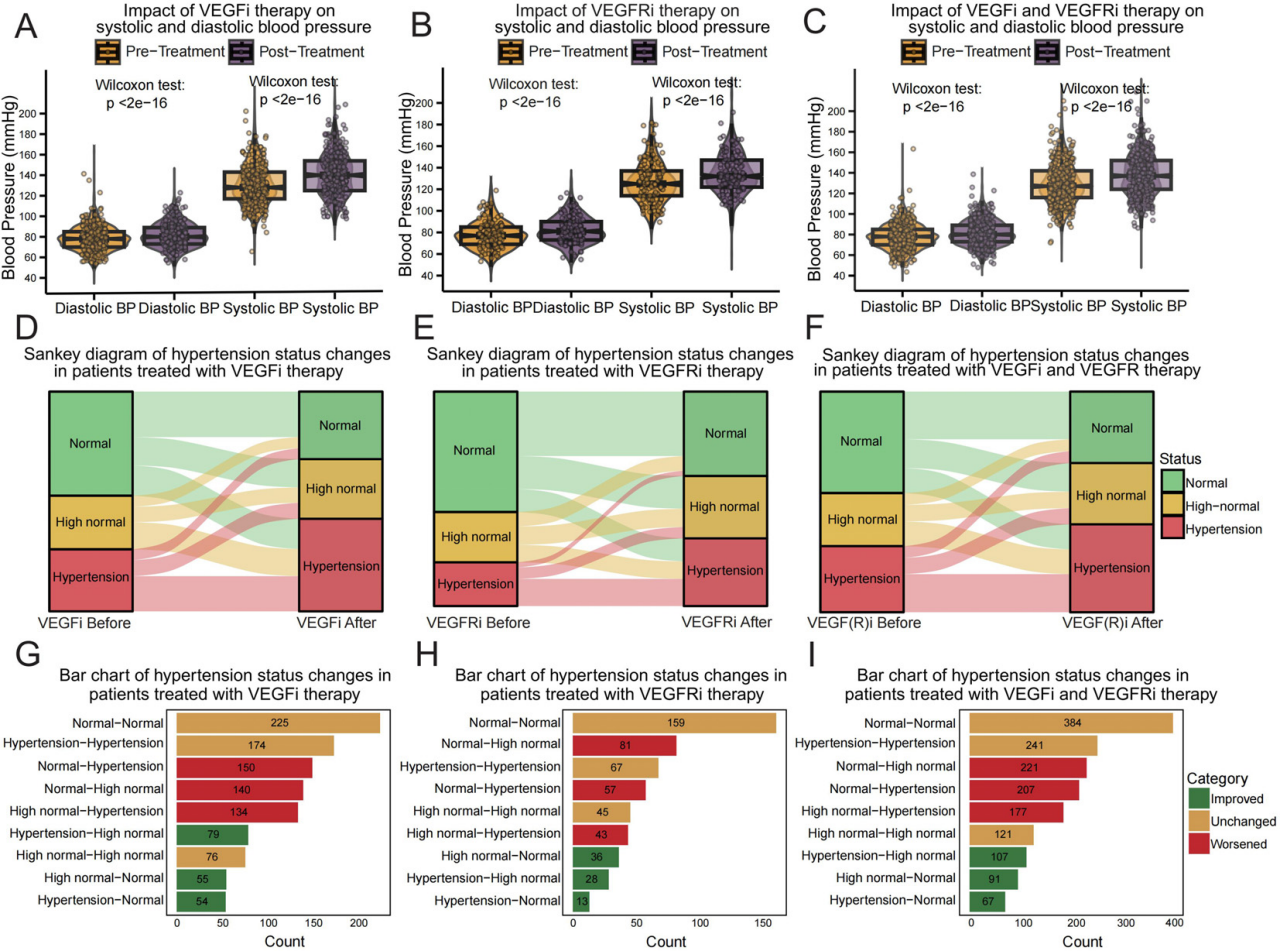
Analysis of blood pressure in clinical patients using VEGFi and VEGFRi. **(A)** Comparative analysis of pre- and post-treatment blood pressure in VEGFi patients: This graph shows the impact on both diastolic and systolic blood pressure in patients receiving VEGFi therapy, highlighting significant increases post-treatment. **(B)** Blood pressure changes post-VEGFRi therapy: This graph displays comparative plots of diastolic and systolic blood pressure before and after VEGFRi treatment, documenting notable shifts towards higher pressures. **(C)** Overall impact on blood pressure by VEGFi or VEGFRi treatment: This graph summarizes the effects on diastolic and systolic blood pressure across the patient cohort treated with both inhibitors. **(D)** Transition dynamics in blood pressure status due to VEGFi: Sankey diagram illustrating shifts in blood pressure categories before and after treatment with VEGFi, visualizing both deterioration and improvements in patient statuses. **(E)** Blood pressure category shifts following VEGFRi administration: This Sankey plot details the changes in blood pressure status for patients treated with VEGFRi. **(F)** Combined effects of VEGFi or VEGFRi on blood pressure status: This Sankey plot summarizes the changes in blood pressure status across the patient cohort treated with both inhibitors. **(G)** Quantitative changes in blood pressure status in VEGFi-treated patients: Bar graph detailing the counts of patients across different hypertension status transitions post-VEGFi therapy. **(H)** Hypertension status changes post-VEGFRi treatment: Bar chart quantifies the transitions in blood pressure status for post-VEGFRi therapy. **(I)** The collective outcomes for patients treated with VEGF or VEGFR inhibitors: Bar chart detailing the predominant trends in hypertension status transitions with both inhibitors.

By evaluating patient data from Zhujiang Hospital of Southern Medical University, we provided insights into the potential effects of VEGFi and VEGFRi on patients’ blood pressure in clinical applications. After performing a comprehensive data analysis, we observed significant increases in both diastolic and systolic blood pressures across different treatment regimes. Specifically, for VEGFi (**Figure 3A**), the median systolic blood pressure increased from 128 mmHg (IQR 117-143) pre-treatment to 140 mmHg (IQR 125-154) post-treatment, and the median diastolic pressure rose from 78 mmHg (IQR 70-85) to 80 mmHg (IQR 73-89). For VEGFRi (**Figure 3B**), the median systolic pressure rose from 125 mmHg (IQR 114-137) to 132 mmHg (IQR 122-147), and the median diastolic pressure increased from 77 mmHg (IQR 69-85) to 81 mmHg (IQR 73-90) after treatment. Statistical tests confirmed that these increases reached significance levels in all treatment groups (P < 2e-16). These results emphasize that the increases in blood pressure were strongly associated with both types of medication, affecting patients regardless of the specific inhibitor used (**Figure 3C**).

In our detailed analysis of hypertension status changes visualized through Sankey diagrams and quantified by bar charts, we observed variations in blood pressure outcomes among patients treated with VEGF and VEGFR inhibitors. The effect of VEGFi (**Figures 3D, G**), where a notable proportion of patients experienced an escalation in blood pressure status: 150 patients transitioned from normal to hypertension and 140 from normal to high normal, indicating a significant worsening. Conversely, a positive shift was observed in 54 patients transitioning from hypertension to normal, with 225 patients remaining normotensive. Similarly, depicting the impact of VEGFRi (**Figures 3E, H**), showed 57 patients progressing from normal to hypertension and 81 to high normal. Here, fewer patients, 13 in total, improved from hypertension to normal, with 159 patients remaining normotensive. When considering the combined effects of both inhibitors (**Figures 3F, I**), the trend towards increasing hypertension was even more pronounced, with 207 patients moving from normal to hypertension and 221 to high normal. Collectively, these findings underscore the substantial impact of VEGFi and VEGFRi on blood pressure.

### 3.4 Effect of VEGFi and VEGFRi on blood pressure in animal models

**Figure 4:**
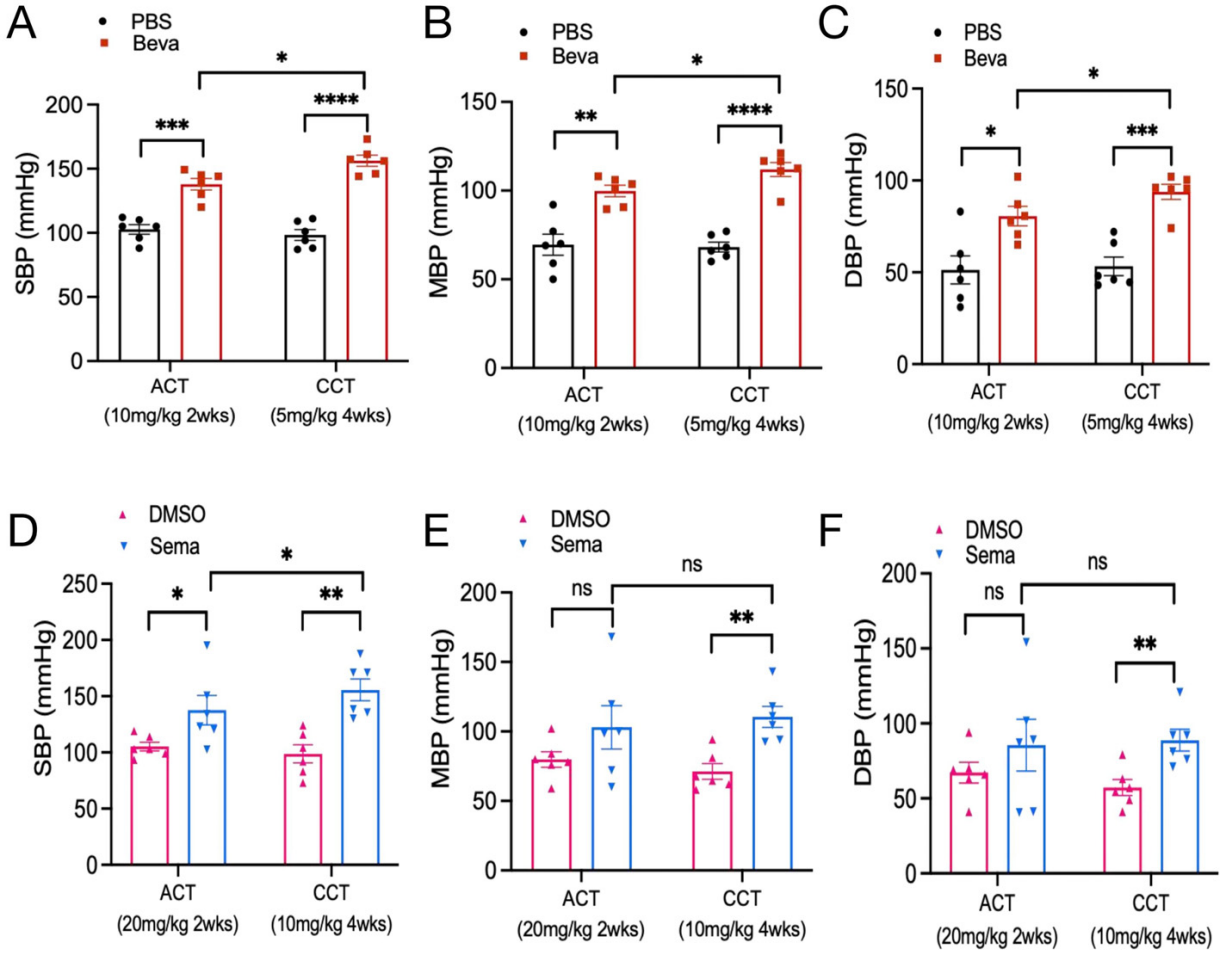
Effect of VEGFi/VEGFRi on blood pressure. **(A-C)**, Effect of VEGFi (Bevacizumab) on SBP **(A)**, MBP **(B)**, and DBP **(C)** at different intervention times; **(D-F)**, Effect of VEGFRi (Semaxanib) on SBP **(D)**, MBP **(E)**, and DBP **(F)** at different intervention times. (Beva=Bevacizumab, Sema=Semaxanib, *p<0.05, **p<0.01, ***p<0.001, ****p<0.0001, ns=not significant).

In animal models, there was no statistically significant difference in body weight at baseline among mice in each group (**Supplementary Figure S2**), and none of the mice died. The BP levels of mice treated with PBS and bevacizumab were examined at 2 and 4 weeks. It was found that compared with the PBS group, the BP levels of mice from the bevacizumab (10 mg/kg) group were dramatically increased after 2 weeks of treatment, where SBP increased by 35.22 ± 5.81 mmHg, MBP increased by approximately 30.32 ± 6.75 mmHg, and DBP increased by approximately 29.23 ± 9.31 mmHg. After 4 weeks of treatment with bevacizumab (5mg/kg) or PBS, the BP in the bevacizumab group was significantly elevated compared to that in the PBS group (ΔSBP: 57.94 ± 6.05 mmHg; ΔMBP: 43.72 ± 4.77 mmHg; ΔDBP: 40.56 ± 6.55 mmHg). Moreover, the SBP/MBP/DBP of mice from the bevacizumab group in the CCT model were significantly higher than those in the ACT model (p<0.05) (**Figures 4A–C**).

Regarding semaxanib, it was found that compared with the DMSO group, semaxanib (20 mg/kg) intervention for 2 weeks caused a sharp increase in SBP of about 32.33 ± 13.74 mmHg (P<0.05). Although there were increases in MBP and DBP, the differences were not statistically significant. In the early BP response induced by semaxanib, SBP would be abnormal and significantly elevated very quickly, but the elevation of MBP and DBP was not significant in some individuals. We further evaluated the chronic cardiotoxicity of semaxanib and found that compared with the DMSO group, mice showed significant elevations in SBP/MBP/DBP after 4 weeks of semaxanib intervention, which were approximately 56.89 ± 12.62 mmHg (P=0.001), 39.25 ± 9.47 mmHg (P=0.002), and 31.47 ± 9.01 mmHg (P=0.006), respectively. Compared with the ACT group, mice in the CCT group showed a sustained increase in SBP, which was significantly higher than that in the ACT group (P<0.05), but there was no statistically significant elevation in MBP and DBP (**Figures 4D–F**).

### 3.5 Acute and chronic effects of VEGFi and VEGFRi on blood pressure

**Figure 5:**
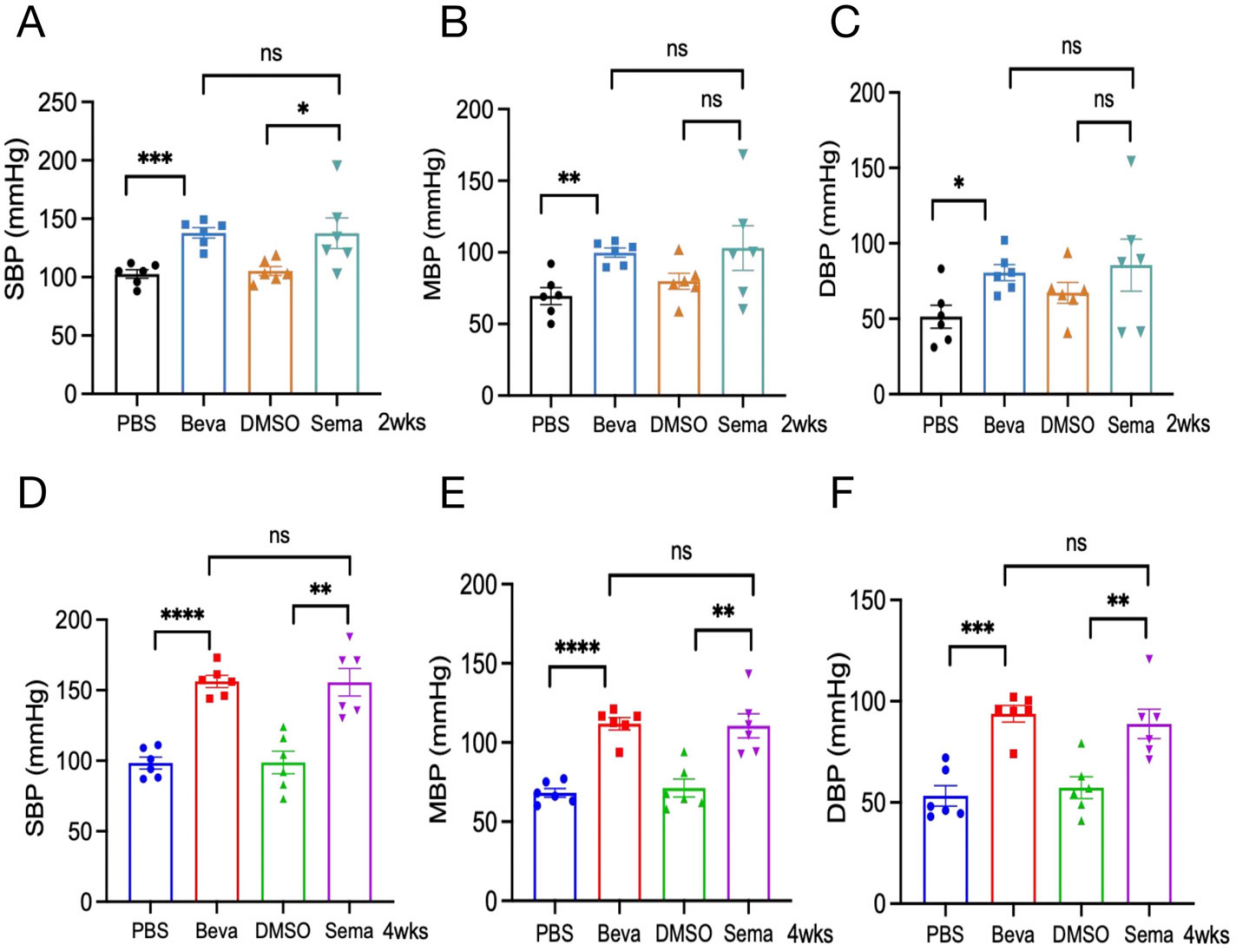
Acute and chronic effects of VEGFi and VEGFRi on blood pressure. **(A-C)** Acute effects of VEGFi and VEGFRi on SBP **(A)**, MBP **(B)**, and DBP **(C)**. **(D-F)** Chronic effects of VEGFi and VEGFRi on SBP **(D)**, MBP **(E)**, and DBP **(F)**. (Beva=Bevacizumab, Sema=Semaxanib, *p<0.05, **p<0.01, ***p<0.001, ****p<0.0001, ns=not significant).

We evaluated the acute and chronic responses of BP levels induced by VEGFi and VEGFRi, indicating that a 2-week intervention of Bevacizumab or Semaxanib both contributed to an increase in BP, with no statistically significant difference (**Figures 5A–C**). Further analysis of chronic cardiotoxicity revealed that there was also no significant difference in SBP/MBP/DBP between animal models treated with Bevacizumab and Semaxanib for 4 weeks (**Figures 5D–F**), suggesting that VEGFi and VEGFRi induced comparable effects on BP responses. Interestingly, we found that the BP of mice in the Bevacizumab groups from the CCT model and ACT model was relatively concentrated. However, the differences in BP among independent samples of mice in the Semaxanib group were very significant, with SBP fluctuating from 102.5 mmHg to 195 mmHg in the Semaxanib group during the acute phase, and from 130 mmHg to 187.67 mmHg during the chronic phase. These outcomes suggested that VEGFRi may have a significant difference in the acute and chronic effects on blood pressure in different individuals compared with VEGFi.

### 3.6 Cross-cancer analysis and discovery of key signaling pathways

**Figure 6:**
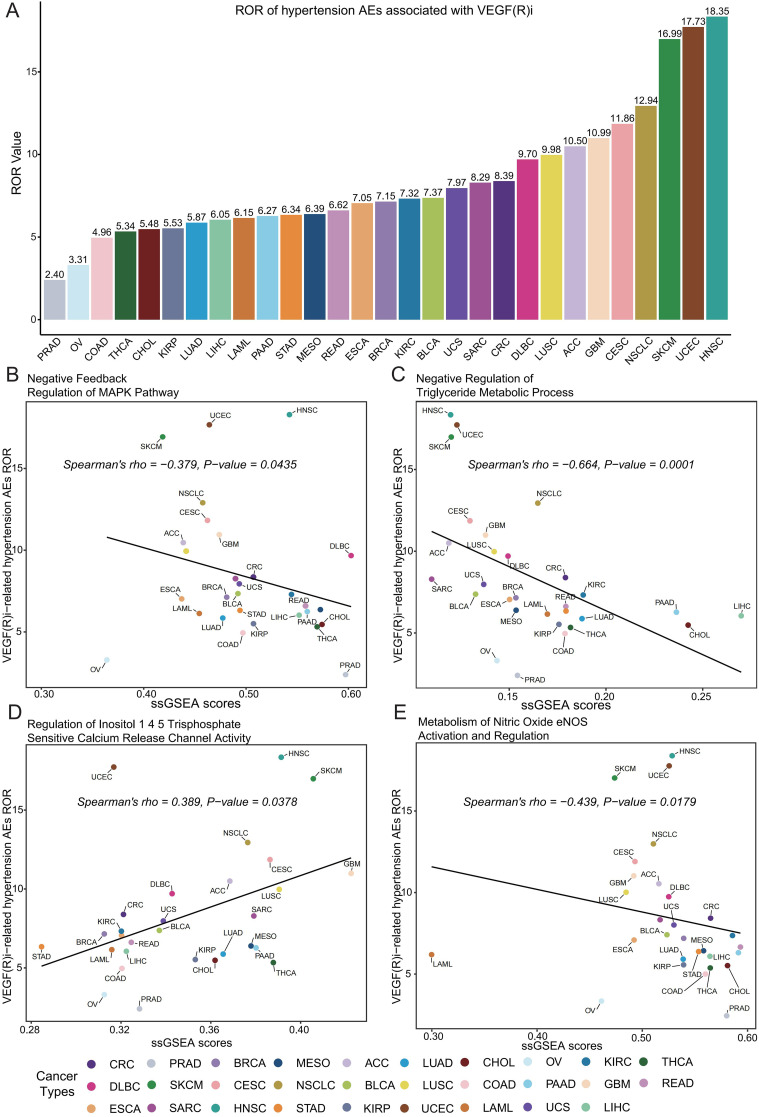
Assessment of the correlation between VEGF(R) inhibitor-associated hypertension adverse effects and key signaling pathways. **(A)** Rates of reporting (ROR) of VEGF(R) inhibitor-associated hypertensive adverse reactions in 29 cancer types; tumor types with fewer than 5 cases were not included in the analysis. **(B)** Correlation analysis of negative feedback regulation of the MAPK pathway with ssGSEA enrichment scores of hypertensive adverse reactions. **(C)** Correlation analysis of negative regulation of triglyceride metabolic processes with ssGSEA enrichment scores of hypertensive adverse reactions. **(D)** Correlation analysis of the regulation of inositol 1,4,5-trisphosphate-sensitive calcium release channel activity with ssGSEA enrichment scores for adverse reactions to hypertension. **(E)** Correlation analysis of nitric oxide eNOS-activated and regulated metabolism with ssGSEA enrichment scores for adverse reactions to hypertension. Cancer types included: PRAD, prostate adenocarcinoma; OV, ovarian cancer; COAD, colon adenocarcinoma; THCA, thyroid cancer; CHOL, cholangiocarcinoma; KIRP, renal pelvis adenocarcinoma; LUAD, lung adenocarcinoma; LIHC, hepatocellular carcinoma; LAML, acute myeloid leukemia; PAAD, pancreatic adenocarcinoma; STAD, gastric adenocarcinoma; MESO, mesothelioma; READ, rectal adenocarcinoma; ESCA, esophageal cancer; BRCA, breast invasive carcinoma; KIRC, kidney clear cell carcinoma; BLCA, bladder urothelial carcinoma; UCS, uterine sarcoma; SARC, soft tissue sarcoma; CRC, colorectal carcinoma; DLBC, diffuse large B-cell lymphoma; LUSC, lung squamous cell carcinoma; ACC, adrenal cortical cancer; GBM, glioblastoma; CESC, cervical squamous cell carcinoma and cervical adenocarcinoma; NSCLC, non-small cell lung cancer; SKCM, skin melanoma; UCEC, endometrial carcinoma; HNSC, head and neck squamous cell carcinoma.

We further explored the association between VEGF(R) inhibitors and hypertension-related adverse effects in the different cancer types examined. Our pan-cancer analysis revealed a significant association between VEGF(R) inhibitors and hypertension-related adverse effects in every cancer type studied (**Figure 6A**). In particular, we observed the highest reported rate in head and neck squamous cell carcinoma (HNSC) (ROR = 18.35, 95% CI [9.54, 35.28]) and the lowest ROR in prostate adenocarcinoma (PRAD) (ROR = 2.40, 95% CI [1.73, 3.33]). By combining the analysis of transcriptomic data from TCGA pan-cancer, we further found that the occurrence of hypertension-related adverse effects was significantly associated with changes in four specific signaling pathways. These included negative feedback regulation of the MAPK pathway (R = -0.379, P = 0.0435) (**Figure 6B**), negative regulation of triglyceride metabolic processes (R = -0.664, P = 0.0001) (**Figure 6C**), modulation of myo-inositol 1,4,5-trisphosphate-sensitive calcium release channel activity (R = 0.389, P= 0.0378) (**Figure 6D**), and nitric oxide eNOS activation and regulation of metabolism (R = -0.439, P = 0.0179) (**Figure 6E**).

## 4 Discussion

Cardiovascular diseases and tumors are the leading causes of death worldwide (42). Growing evidence has suggested a strong interrelationship between the two main causes, with an elevating global healthcare burden. With the widespread use of VEGF pathway inhibitors in cancer patients, drug-induced cardiovascular disease-related adverse effects have a serious impact on the prognosis of cancer patients. The most common cardiovascular adverse effects caused by VEGF pathway inhibitors are hypertension, which is considered to be associated with an imbalance in vasoconstrictor-diastolic homeostasis (43), structural changes in the microvasculature (44), and an increase in oxidative stress (45). Few studies have focused on whether there are differences in the time to onset of hypertension and the degree of blood pressure elevation caused by different drugs of VEGF pathway inhibitors. Therefore, we analyzed the risk and onset time of hypertension caused by different VEGF and VEGFR inhibitors in the FAERS database and verified the findings using clinical data. Subsequently, animal experiments were conducted to verify the differences in the onset time of BP elevation and the degree of hypertension caused by VEGF and VEGFR inhibitors. Lastly, cross-cancer analysis was conducted to reveal the key signaling pathways of VEGFi/VEGFRi-induced hypertension.

ROR and IC are used to quantify the association between hypertension and the use of VEGF and VEGFR inhibitors, while also considering the reduction of false correlations driven by reporting biases or data anomalies. ROR provides a measure of the relative odds of an adverse event occurring with the use of a drug compared to not using the drug. On the other hand, IC reflects direct clinical relevance, providing a logarithmic measure to assess how frequently a specific adverse event occurs with a drug compared to what would be expected under independent conditions, adjusted for the total number of reports. In our study, if both the ROR and IC for specific hypertension-related adverse reactions associated with VEGF or VEGFR inhibitors are significant, it indicates a robust link between drug usage and the reported adverse events.

Data from the FAERS database, combined with clinical profiles, confirmed that VEGF and VEGFR inhibitors significantly increase the risk of hypertension-related adverse events (46). Animal experiments revealed that short-term intervention with VEGF and VEGFR inhibitors could lead to BP elevation, and data from the CCT models compared with ACT models showed that lasting therapy with VEGF and VEGFR inhibitors could lead to a continuous increase in BP. This suggests that the body may have a certain tolerance to the cardiotoxicity induced by VEGF and VEGFR inhibitors, but the elevated BP effects continue to accumulate time-dependently, even if the dosage is reduced by half. Thus, we found that VEGF(R) inhibitors were not only dose-dependent (22) but also significantly time-dependent, and the latter effect was significantly greater than the former. Therefore, long-term continuous monitoring of BP for patients treated with VEGF(R)i is required, regardless of whether the regimen is adjusted or the dosage is reduced.

We found that different inhibitors of the VEGF pathway showed different tendencies in inducing specific BP abnormalities, where VEGF inhibitors showed more positive signals for blood pressure-related adverse reactions compared to VEGF inhibitors. Additionally, there were significant differences in the overall time to onset of hypertension caused by VEGFi and VEGFRi. Meanwhile, VEGFRi showed a significantly shorter overall time to onset of hypertension and hypertension-related adverse events than VEGFi. There were also significant differences in the time to onset of hypertension due to different drugs, with Bevacizumab showing a significantly longer onset time relative to other VEGFR inhibitors. Further experimental animal studies verified that there was significant heterogeneity in the BP response induced by Semaxanib (a VEGFRi) when compared with Bevacizumab. This suggested that some cancer patients experienced a sharp elevation in BP (SBP>180 mmHg), unlike the smooth increase of VEGFi, which significantly increased the risk of hypertensive encephalopathy, cerebral hemorrhage, and other acute cardiovascular events in patients. This difference may be related to SNP polymorphisms in the gene encoding VEGFR in cancer patients (47, 48). Therefore, early and close monitoring of blood pressure and timely antihypertensive treatment are more necessary for VEGFRi-treated patients. Meanwhile, hypertension induced by VEGFi and VEGFRi may serve as a biomarker of treatment tumor effects and patient prognosis (49). Several clinical reports have indicated that the overall survival (OS) and progression-free survival (PFS) of hypertensive patients caused by VEGF(R)i are significantly higher than those of patients with normal blood pressure and are significantly positively correlated with blood pressure (50, 51), which may be associated with tumor vascular hypersensitivity (52, 53). Animal models proved that both Bevacizumab and Semaxanib could lead to hypertension in both acute and chronic treatment, and the VEGFi/VEGFRi-induced elevated BP effect was comparable, which may suggest that the overall therapeutic effect of VEGFi and VEGFRi on tumors is comparable. Additionally, the anti-cancer effect of VEGFRi generally appeared earlier for a sharp elevation of BP, but the effect of VEGFRi may be suboptimal in some patients due to the significant heterogeneity of the BP response.

The mechanisms related to the development of hypertension caused by VEGFi and VEGFRi in patients with various tumors were further explored, and it was found that the MAPK pathway showed significant negative feedback regulation after treatment with VEGFi and VEGFRi (R = -0.379, P = 0.0435). The MAPK pathway, by promoting the proliferation, differentiation, migration, and apoptosis of vascular endothelial cells, exerts a significant impact on vascular function and structure (54). Interestingly, it showed a significant negative regulation of triglyceride metabolic processes (R = -0.664, P = 0.0001) which implies the imbalanced lipid metabolism and increased lipid accumulation. Since lipids and their metabolites are increasingly recognized as key players in complex signaling pathways, abnormalities in lipid metabolism and accumulation of lipids due to VEGFi/VEGFRi can modulate the immune responses in a variety of ways, including lipid metabolite responses to pathogens, phagocytosis and inflammation. At the same time, abnormal lipid metabolism can lead to lipid peroxidation, which promotes vascular endothelial damages. It is a risk factor for adverse cardiovascular events such as hypertension (55–57). A recent study has showed that immunobiomaterials can attenuate local inflammation in tumors by modulating the function of immune cells, and that they may reduce the infiltration of vascular endothelial inflammatory cells and endothelial damage caused by VEGFi/VEGFRi (58). Regarding the activity of inositol 1,4,5-trisphosphate-sensitive calcium release channels, an enhancement was observed (R = 0.389, P = 0.0378), revealing enhanced Ca(2+) signaling and increased responsiveness to vasoconstrictors in the vascular smooth muscle, providing evidentiary support for the rise in blood pressure (59). As for the metabolic pathways activated and regulated by nitric oxide eNOS, they showed a tendency to be suppressed (R = -0.439, P = 0.0179). Considering the central role of nitric oxide (NO) in the regulation of blood flow and blood pressure, the downregulation of eNOS led to a significant reduction in the vasodilatory response of vascular endothelial cells, which further contributed to the increase in systolic and diastolic blood pressure (60).

Early monitoring and effective control of blood pressure in oncology patients with VGEFi/VEGFRi as VEGFi/VEGFRi can rapidly lead to an increase in blood pressure. In addition to conventional blood pressure monitoring, nanomaterial-assisted metabolic profiling can be used to monitor biological markers in the patient’s blood for early diagnosis and to assist in treatment (61). Meanwhile, for people with VEGFi/VEGFRi-induced hypertension, conventional antihypertensive regimens can be utilized, including CCBs, ACEIs, and ARBs used singly or in combination. And now there are scholars using different methods to quantify the dose of antihypertensive drugs and analyze and record the dose of drug combinations, which is more accurate for monitoring the use of different antihypertensive drugs to help the effective control of blood pressure (62–64). Therefore, the rational selection and concentration monitoring of antihypertensive drugs in VEGFi/VEGFRi-treating patients could help to reduce the incidence of adverse cardiovascular events and effectively improve cardiovascular mortality in this subset of tumor patients.

The following limitations exist in this study: 1) We confirmed that there were significant differences in the time of blood pressure rise between different VEGFi and VEGFRi drugs through the FAERS database, clinical data, and animal experiments. The animal study found that the degree of blood pressure rise in the early stage of VEGFRi was significantly higher than that of VEGFi, but with a large degree of dispersion, which needs to be supported by more preclinical and clinical data. 2) Clinical data have not been available on VEGFi and VEGFRi cancer patients with hypertension grading and staging, and the risk of acute cardiovascular events such as hypertensive encephalopathy and cerebral hemorrhage caused by VEGFi and VEGFRi needs to be assessed to further evaluate the clinical role and risk of different VEGF pathway inhibitors.

## 5 Conclusions

In this study, we demonstrated that VEGFi and VEGFRi significantly increased the risk of blood pressure-related adverse events, with the time-dependent effect being significantly greater than the dose-dependent effect. We also found that the onset of hypertensive events resulting from VEGFRi treatment was earlier, and the short-term sharp increase in BP was more pronounced, and it showed more positive signals for blood pressure-related adverse reactions compared to VEGFi. Therefore, long-term blood pressure monitoring should be performed in VEGFi and VEGFRi-treated cancer patients. Especially in VEGFRi patients, BP levels should be monitored as early as possible, which can help reduce the risk of other cardiovascular complications including heart failure, stroke, arrhythmia, and myocardial infarction, and further improve the long-term prognosis of cancer patients.

## Data Availability

The original contributions presented in the study are included in the article/**Supplementary Material**. Further inquiries can be directed to the corresponding authors.

## Supplementary material

Supplementary Figure 1 Comparative analysis of time to onset of blood pressure-related adverse reactions of specific VEGFi vs. VEGFRi.

Supplementary Figure 2 Body Weights of Mice at Baseline. **(A)** Body weights of different groups of mice in the ACT groups. **(B)** Body weights of different groups of mice in the CCT groups.

Supplementary Table 1 The standard and trade names for VEGFi and VEGFRi, ensuring accuracy and consistency in patient data retrieval mentioned in the text.

Supplementary Table 2 A list of hypertension-related adverse reactions included in the study, ensuring the accuracy of risk analysis for patients using VEGFi or VEGFRi discussed in the text.

Supplementary Table 3 The results of positive signal detection for hypertension-related adverse reactions, treating VEGF and VEGFR inhibitors as a collective entity.
