# Supplementary figures and images for: Comprehensive analysis of VEGF/VEGFR inhibitor-induced immune-mediated hypertension: integrating pharmacovigilance, clinical data, and preclinical models
Source: Front Immunol. 2024 Oct 22;15:1488853. doi: 10.3389/fimmu.2024.1488853 (PMC11534862; doi:10.3389/fimmu.2024.1488853)

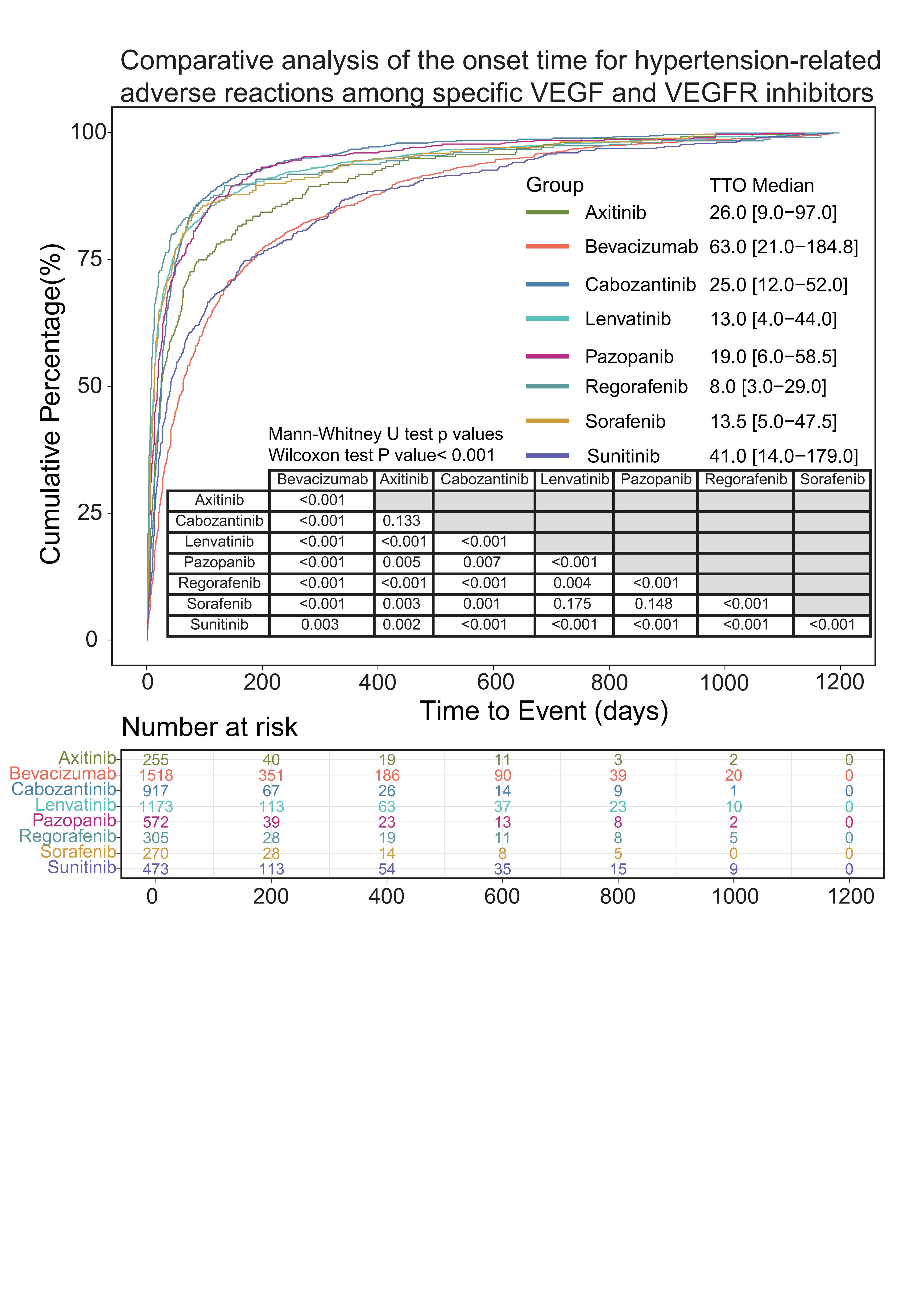

Supplement: Supplementary Figure 1 — Comparative analysis of time to onset of blood pressure-related adverse reactions of specific VEGFi vs. VEGFRi. [file Image1.jpeg]

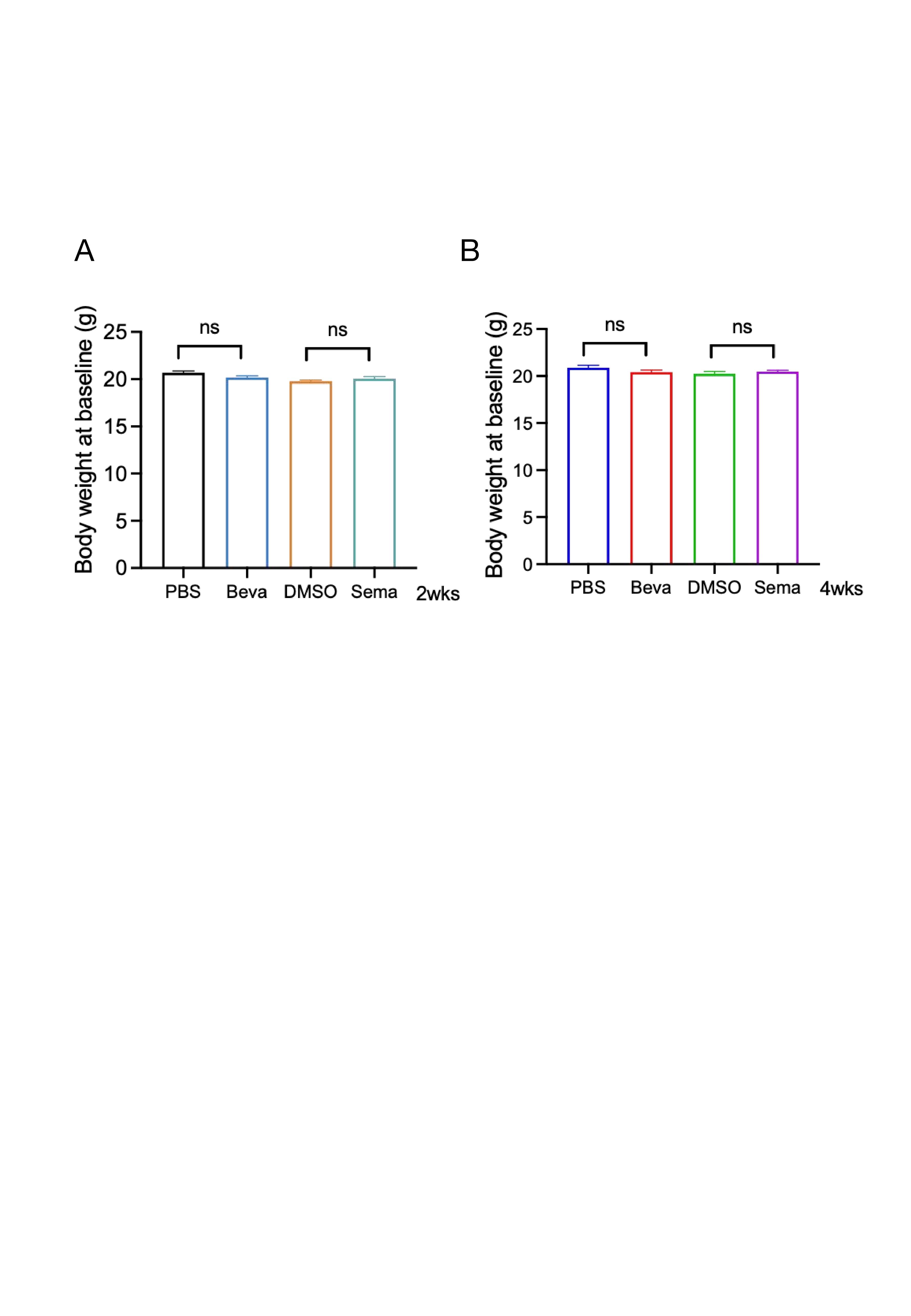

Supplement: Supplementary Figure 2 — Body Weights of Mice at Baseline. (A) Body weights of different groups of mice in the ACT groups. (B) Body weights of different groups of mice in the CCT groups. [file Image2.jpeg]
